# Transcranial direct current stimulation over the frontal eye field has no effect on visual search performance

**DOI:** 10.1093/braincomms/fcaf480

**Published:** 2025-12-08

**Authors:** Charline Peylo, Constanze Albrecht, Ruth Gerlinde Homann, Joshua Richter, Marie Anaïs Dornier, Mona Sophie Ehrat, Finia Luca Loeb, Sophia Manz, Marina Praguer Gaeta, Christina Rieger, Julia Christine Tafelmaier, Paul Sauseng

**Affiliations:** Neuropsychology and Cognitive Neuroscience, Department of Psychology, University of Zurich, Zurich 8050, Switzerland; Biological Psychology, Department of Psychology, Ludwig-Maximilian University Munich, Munich 80802, Germany; Biological Psychology, Department of Psychology, Ludwig-Maximilian University Munich, Munich 80802, Germany; Biological Psychology, Department of Psychology, Ludwig-Maximilian University Munich, Munich 80802, Germany; Biological Psychology, Department of Psychology, Ludwig-Maximilian University Munich, Munich 80802, Germany; Biological Psychology, Department of Psychology, Ludwig-Maximilian University Munich, Munich 80802, Germany; Biological Psychology, Department of Psychology, Ludwig-Maximilian University Munich, Munich 80802, Germany; Biological Psychology, Department of Psychology, Ludwig-Maximilian University Munich, Munich 80802, Germany; Biological Psychology, Department of Psychology, Ludwig-Maximilian University Munich, Munich 80802, Germany; Biological Psychology, Department of Psychology, Ludwig-Maximilian University Munich, Munich 80802, Germany; Biological Psychology, Department of Psychology, Ludwig-Maximilian University Munich, Munich 80802, Germany; Biological Psychology, Department of Psychology, Ludwig-Maximilian University Munich, Munich 80802, Germany; Neuropsychology and Cognitive Neuroscience, Department of Psychology, University of Zurich, Zurich 8050, Switzerland; Neuroscience Center Zurich, University of Zurich, Zurich 8057, Switzerland

**Keywords:** top-down attention, visual-spatial attention, visual search, frontal eye field, transcranial direct current stimulation

## Abstract

Top-down attention (i.e. the goal-directed (de-)prioritization of information) is fundamental for successful everyday life. Attention deficits caused by brain lesions, like visuospatial neglect or extinction, are therefore of major importance and call for effective therapies. Transcranial direct current stimulation (tDCS), a non-invasive, electric brain stimulation technique, has been discussed as a potential therapeutic tool. Recent research suggests that anodal tDCS over the frontal eye field (FEF) might increase visual search performance even in healthy participants, substantiating the potential therapeutic efficacy of tDCS (e.g. for stroke rehabilitation). In two pre-registered experiments, we investigated the robustness of these findings. In the first experiment, the right FEF was anodally stimulated, supposedly increasing neural activity; in the second experiment, anodal tDCS was delivered over the left FEF, and the size of the visual search field was manipulated. In neither of the two experiments, previous findings of enhanced visual search performance due to tDCS could be reproduced. In contrast, Bayesian statistics indicated evidence against reliable top-down attention-guided visual search improvements through FEF tDCS in healthy participants. Although effects might be stronger in patient populations, the present results do not suggest tDCS over FEF to be a very strong candidate as a therapeutical approach in attention disorders.

## Teaser

Peylo *et al*. (2025) report a series of experiments which probed visual search improvements through transcranial direct current stimulation over the frontal eye fields. Their results provide no evidence for stimulation-specific visual search improvements or behavioural baseline dependencies, challenging the generalizability and broader therapeutic potential of this specific stimulation approach.

## Graphical Abstract

**Graphical Abstract:**
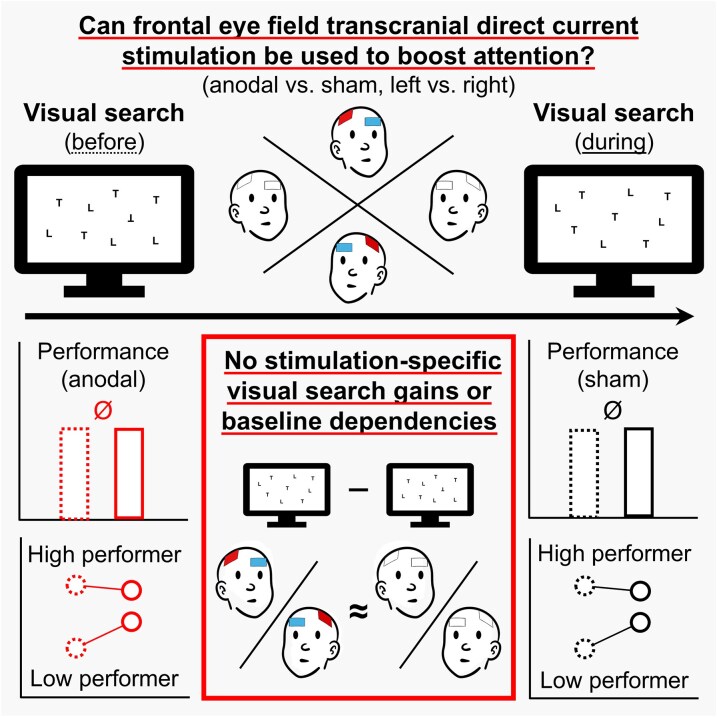

## Introduction

Top-down attention, the voluntary (de-)prioritization of information like the selection of certain visual sub-fields^1^ controlled by a dorsal fronto-parietal network including the frontal eye field (FEF),^2^ is central to successful everyday-behavior.^3^ Patients with brain lesions suffering from attention deficits like visuospatial neglect or extinction are therefore strongly impaired in their day-to-day life and the quality of such.^4^ This relevance makes attention a key target for stimulation-based enhancement and rehabilitation attempts in healthy and clinical populations, respectively.^5,6^ Transcranial direct current stimulation (tDCS) has been proposed as safe and easy tool for non-invasively manipulating neural excitability (and thereby possibly cognition) through the scalp-level application of weak electric currents.^7^ In line with this, a recent study reported significant visual search improvements through anodal (excitatory) versus sham (control) tDCS over the left FEF^8^ even in healthy participants, highlighting the therapeutic potential of tDCS for attention improvements within and beyond clinical settings (e.g. for post-stroke rehabilitation). This effect was inversely related to baseline performance (i.e. larger stimulation-specific improvements were observed for participants who performed low at baseline).^8^

Based on the proposed dominance of the right FEF for visual-spatial top-down attention,^9^ in a first experiment, we tested whether the previously reported visual search improvement^8^ could be reproduced and potentially further augmented through right instead of left FEF stimulation. Moreover, in a second experiment, we probed the potential dependence of such effect on search field size. We hypothesized that tDCS might only improve performance when searching large visual fields that require greater spatial attention shifts and longer saccades, thereby potentially reinforcing visual search improvements through FEF stimulation. To these ends, in both experiments, we asked participants to perform a visual search for a target letter within small or large search fields before (baseline) and during (peri) the application of sham or anodal tDCS over the right or left FEF.

## Materials and methods

### Participants

A preregistered *a-priori* power analysis in G*Power^©^ (v3.1.9.4; Heinrich Heine University, Düsseldorf, Germany^10^) for a one-tailed paired-sample *t-*test with an estimated effect size of Cohen’s *d* = 0.5 (as conservative approximation of effect sizes reported by previous studies with FEF tDCS effects on different visual search outcomes^8,11^), an alpha level of α = 0.05 and a desired power of 1-β = 0.80 indicated a required sample size of *N* = 27. Based on this estimate and a potential drop-out of participants over the course of our two-session experiments, we tested *N* = 29 and *N* = 32 healthy participants in Experiments 1 and 2, respectively. In contrast to our preregistered policy of no performance-based participant exclusions, we excluded one participant from Experiment 2 *post-hoc* due to unreasonably low scores across all conditions of the sham session (all <22% correct), suggesting a potential confusion of the stimulus-response mapping. The final sample of Experiments 1 and 2 therefore consisted of *N* = 29 [*M_age_* = 21.62, *SD_age_* = 1.82; female = 15 (52%), male = 12 (41%), diverse = 2 (7%)] and *N* = 31 participants [*M_age_* = 21.48, *SD_age_* = 2.31; female = 22 (71%), male = 9 (29%), diverse = 0 (0%)], respectively.

All participants were right-handed according to the Edinburgh Handedness Inventory,^12^ reported normal or corrected-to-normal vision and no history of neurological or psychological disorders. They further fulfilled all tDCS safety inclusion criteria by confirming absence of the following: diagnosis or close family history of epilepsy, traumatic brain injuries, concussions/blackouts, tinnitus, pregnancy, metallic implants or cochlear/neural/cardiac stimulators, excitability-increasing medication, spinal cord surgery or cerebrospinal puncture, sleep deprivation or drug/increased alcohol intake within 24 h before the experiment. Participants gave their written informed consent at experiments’ beginnings and could receive course credits as compensation at their ends. The studies were approved by LMU’s F11 ethics committee (ethics approval reference number ‘02_2022_Sauseng_a’ from April 6th 2022) and were performed according to laws and the declaration of Helsinki.

### Visual search task

**Figure 1:**
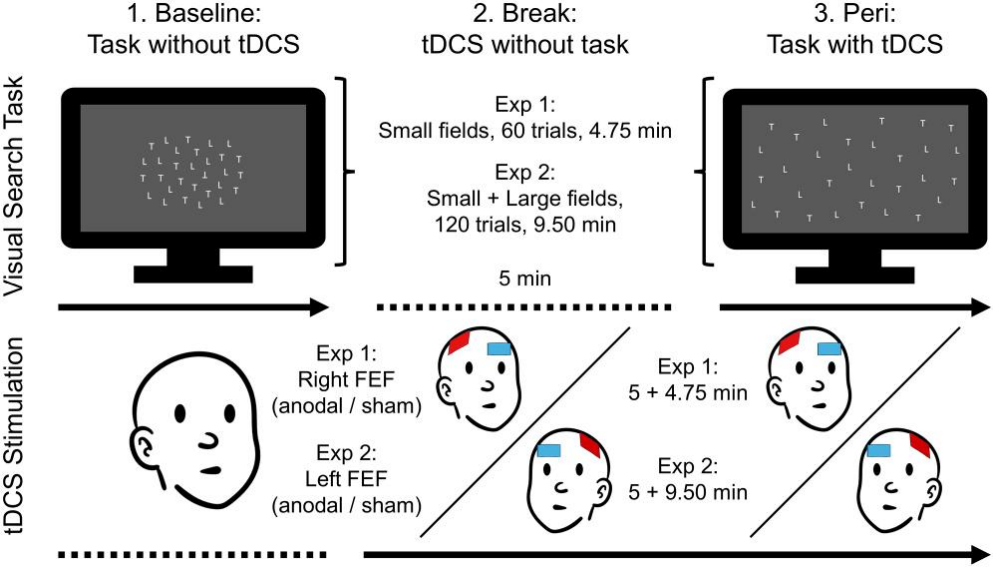
**Visual search task and experimental procedure.** In two experiments, *N* = 29 and *N* = 31 participants performed a visual search task (upper panel), in which they indicated the presence (left example stimulus) or absence (right example stimulus) of an upside-down ‘T’ amongst upright ‘T’s and ‘L’s. This task was performed twice, once before (baseline; lower left panel) and once during (peri; lower right panel) the application of transcranial direct current stimulation (tDCS) over the frontal eye field (FEF). In between, 5 min of passive tDCS were applied (i.e. stimulation in the absence of any sensory information or task; lower middle panel) to allow potential stimulation-driven excitability changes to unfold before task execution. The entire procedure was repeated across two separate sessions, once with anodal tDCS and once with sham tDCS (order counterbalanced). In all sessions, in Experiment 1, the right FEF was stimulated and participants searched exclusively small visual fields (left example stimulus), whereas in Experiment 2, the left FEF was stimulated and participants searched not only small but also large visual fields (right example stimulus). *Note: Stimuli in this figure have been increased in size for illustration purposes.*

All experiments were preregistered, and data and analyses were provided (preregistration and materials can be found at https://osf.io/f64h7). The visual search task and experimental procedure are depicted in Fig. 1. The task was presented on a standard laptop using Presentation® (v0.7; Neurobehavioral Systems^©^, Berkeley, CA, USA) and consisted of a visual search field (1750 ms; 3000 ± 500 ms inter-trial interval), comprising a total of 30 letters (0.4° visual angle height each): 15 upright ‘L’s, 14 upright ‘T’s and either an upside-down ‘T’ (target present trial, 50%) or an additional upright ‘T’ (target absent trial, 50%). The participants’ task was to freely explore the visual search field (i.e. without restriction of eye movements) and to indicate target presence or absence via button press as quickly and precisely as possible (‘Y’/‘N’ using the index finger of the left/right hand, respectively).

The visual search task in Experiments 1 and 2 only differed with respect to search field size and trial count/task duration: In Experiment 1, the search field was always small (7.2°×7.2° visual angle), and each assessment (baseline/peri) consisted of one single block with 60 trials (30 target absent/present trials; conditions randomly interleaved) and lasted about 4.75 min, adding up to a total duration of about 35 min per session (sham/anodal), including training (20 trials) and tDCS preparation. In Experiment 2, only half of the trials contained a small search field (7.2°×7.2° visual angle), whereas the other half of trials contained a large search field (21.3°×13.1°). To account for the additional field size condition while maintaining comparability with the first experiment, here, each assessment (baseline/peri) consisted of one single block with 120 trials (30 target present/absent in small/large search field trials; conditions randomly interleaved) and lasted about 9.5 min, adding up to a total duration of about 50 min per session (sham/anodal), including training (40 trials) and tDCS preparation.

### tDCS stimulation

The stimulation protocol closely resembled that of Gan *et al*.^8^ and was identical for the two experiments, with the notable exception that the first experiment used right FEF stimulation to probe the presumed right-hemispheric dominance of top-down attention,^9^ whereas the second experiment used left FEF stimulation to increase comparability with previous studies^8^ and thereby exclude right FEF stimulation in Experiment 1 as potential explanation for possible null-effects. In both experiments, stimulation via constant current was delivered using a TCT Research Limited (Hong Kong, China) tDCS device. Positioning of the two saline-soaked sponge electrodes (5 cm × 5 cm each) closely mirrored that of Gan *et al*.^8^ with the anode placed over the FEF (4 cm anterior and 5 cm right/left lateral to the vertex for right/left FEF stimulation in the first versus second experiment, respectively) and the cathode placed above the opposite eye. For the anodal stimulation, current was ramped up to 2 mA (20 s), maintained at that level for the duration of the visual search task and then ramped back down to 0 mA (20 s). For the sham stimulation, current ramped up to 2 mA (20 s) and then immediately ramped back down to 0 mA (20 s) (similar to Gan *et al*.^8^). The two types of stimulation (sham/anodal) were delivered in two separate single-blinded sessions at least 24 h apart with session order counterbalanced across participants.

Importantly, whereas in the previous study the visual search task was started immediately at stimulation onset and performance during the first 5 min was used as an implicit baseline under the assumption of delayed stimulation effects, in the present study, the visual search task for the peri assessment only started 5 min into the stimulation (i.e. after 5 min of passive stimulation according to the above protocol, but during quiet wakefulness in the absence of any sensory information or task), while baseline performance was explicitly assessed in a separate block of the visual search task. This allowed us not only to let potential stimulation-driven excitability changes unfold before task execution (similar to Gan *et al*.^8^) but also to properly account for possible baseline-/state-dependent tDCS effects.^13,14^

### Stimulation blinding

To assess blinding success (and hence participant expectations and potential response biases), at the end of both experiments’ second session, participants were asked to assign stimulation types to test sessions (in Experiments 1 and 2) and to indicate expected stimulation effects (excitation/inhibition; in Experiment 2 only).

### Statistical analysis

Task performance in each condition (sham/anodal, baseline/peri, small/large search fields) was computed in Excel (Microsoft®, Redmond, WA, USA) and later statistically analysed in Jamovi (v2.3.28)^15^ as baseline-corrected percentage correct (percentage correct during stimulation minus percentage correct before stimulation, where each trial with a matching first response was considered correct) rather than as absolute values (such as in Gan *et al*.^8^) to account for inter-individual behavioural differences at baseline.^13,14^ To test our first hypothesis of larger stimulation-related visual search improvements through anodal versus sham right FEF tDCS, in Experiment 1, we computed a preregistered one-tailed paired-sample *t*-test (including Bayesian equivalent) with stimulation (anodal versus sham) as independent variable and baseline-corrected percentage correct as dependent variable. Similarly, to test our second hypothesis of search field size as potential moderating factor of tDCS-driven visual search improvements (and, hence, our expectation of greater visual search improvements through anodal versus sham left FEF tDCS for large but not small search fields), in Experiment 2, we repeated the above *t-*test (and Bayesian equivalents) separately for small and large visual fields. This approach was preregistered and chosen because it not only best captured the essence of our hypothesis but also because it allowed us to maximize comparability between the results of the first and the second experiment.

In a second step, we tested whether potential tDCS-related visual search improvements were negatively related to baseline performance^8^ and whether this effect was specific to anodal stimulation. To this end, we computed non-parametric, one-tailed Spearman rank correlations (accounting for non-normality and/or outliers in some of the relevant variables) between performance at baseline and performance changes from pre- to post-stimulation. This was done separately for each stimulation condition (anodal versus sham) and search field size (small versus large). Bonferroni correction was used to account for multiple correlation testing and (corrected) *P*-values were considered statistically significant if *P* < 0.05.

In a last step, we performed three control analyses. The first two controls repeated the above analyses with signal detection parameters sensitivity (*d’*) and false alarm rates as alternative dependent measures to exclude potential influences of variable choices on analysis outcomes. The third control was a joint analysis of the common small-field condition from both experiments to leverage on the full data spectrum and to compare stimulation sites (left versus right FEF) more systematically (for details see Supplementary material). Results were visualized in PowerPoint (Microsoft®, Redmond, WA, USA) and the Spyder environment (v5.4.3)^16^ for Python (v3.11.3)^17^ using custom-written scripts and the open-source packages NumPy (v1.24.3)^18^ and Matplotlib (v3.7.1).^19^

## Results

### tDCS effects on visual search performance

**Figure 2:**
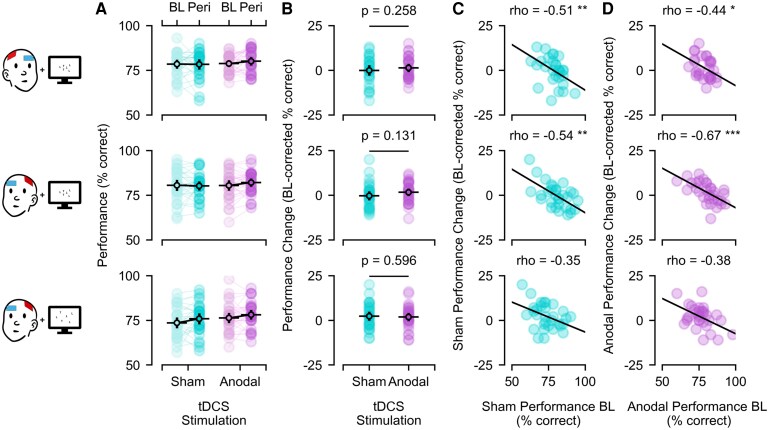
**Visual search performance across tDCS stimulation sites and search field sizes.** (**A**) Visual search performance before (baseline, BL) and during (peri) sham (turquoise) or anodal (purple) frontal eye field (FEF) transcranial direct current stimulation (tDCS), **(B)** stimulation-related performance changes relative to baseline and **(C, D)** their association with baseline performance separately for sham and anodal tDCS, respectively. Rows relate to the different experiments and conditions (row one: Experiment 1 (*N* = 29), right FEF tDCS, small search fields; row two: Experiment 2 (*N* = 31), left FEF tDCS, small search fields; row three: Experiment 2 (*N* = 31), left FEF tDCS, large search fields). Coloured dots represent single-subject data, whereas black circles with horizontal bars represent sample means with 95% confidence intervals (vertical bars). *Note: Electrode setups and search fields were simplified for illustration purposes; P−/rho-values refer to the respective t/Spearman test results with asterisks indicating statistical significance, where* * ≙ *P* < 0.05, ** ≙ *P* < 0.01 and *** ≙ *P* < 0.001.

The results of experiment one are depicted in row one of Fig. 2. Although there was good overall task performance without ceiling effect (sham-baseline: *M* = 78.41%, *SD* = 6.74%; sham-peri: *M* = 78.28%, *SD* = 7.64%; anodal-baseline: *M* = 78.69%, *SD* = 5.19%; anodal-peri: *M* = 80.00%, *SD* = 6.33%), the preregistered *t-*test was not significant [sham: Δ*M* = −0.14%, Δ*SD* = 7.71%; anodal: Δ*M* = 1.31%, Δ*SD* = 6.18%; *t*(28) = 0.66, *P* = 0.258, *d* = 0.122, 95% CI = (−0.244, 0.487)]. Exploratory Bayesian statistics demonstrated about three times more evidence for *H_0_* over *H_1_* (*BF_10_^+^* = 0.35). Moreover, while the previous finding of an inverse relationship between baseline performance and tDCS-related changes^8^ could be reproduced in exploratory correlation analyses of the anodal condition (ρ(27) = −0.44, *p_Bonf_* = 0.017), the same was true for the sham condition (ρ(27) = −0.51, *p_Bonf_* = 0.005). These results were comparable when sensitivity *d’* or false alarm rates were used as alternative dependent variables (for details see Supplementary material). Using right FEF tDCS our first experiment could, thus, neither boost nor reproduce the previously reported visual search improvement following left FEF tDCS^8^ and suggests that apparent stimulation-related effects (improvements for low baseline performers and vice versa) might reflect rather stimulation-unspecific effects (e.g. regression to the mean).

However, one could argue that (in contrast to our hypothesis and previous literature^9^) right FEF tDCS might have been less suitable for this task than the previously used left FEF stimulation.^8^ Moreover, compared to the original study,^8^ we used rather small search fields (7.2° visual angle versus whole-screen presentations), which might require smaller spatial attention shifts and shorter saccades, thereby potentially limiting visual search improvements through FEF stimulation. In contrast to this possibility, however, the results of our second experiment with left FEF stimulation and large search fields confirmed the null-effect of Experiment 1.

Results are depicted in rows two (small fields) and three (large fields) of Fig. 2. In line with Experiment 1, performance was overall good without ceiling effects, but again largely comparable across tDCS sessions and test times, for both small search fields (sham-baseline_small_: *M* = 80.55%, *SD* = 8.50%; sham-peri_small_: *M* = 80.23%, *SD* = 7.05%; anodal-baseline_small_: *M* = 80.45%, *SD* = 7.96%; anodal-peri_small_: *M* = 82.10%, *SD* = 6.41%) and large search fields (sham-baseline_large_: *M* = 73.55%, *SD* = 8.21%; sham-peri_large_: *M* = 75.81%, *SD* = 8.46%; anodal-baseline_large_: *M* = 76.29%, *SD* = 7.80%; anodal-peri_large_: *M* = 78.10%, *SD* = 7.30%). Accordingly, the preregistered *t-*tests were again not significant, not only for small fields as hypothesized [sham_small_: Δ*M* = −0.32%, Δ*SD* = 6.92%; anodal_small_: Δ*M* = 1.65%, Δ*SD* = 5.82%; *t*(30) = 1.14, *P* = 0.131, *d* = 0.205, 95% CI = (−0.152, 0.559)], but also for large fields [sham_large_: Δ*M* = 2.26%, Δ*SD* = 7.03%; anodal_large_: Δ*M* = 1.81%, Δ*SD* = 6.35%; *t*(30)=−0.25, *P* = 0.596, *d* = −0.044, 95% CI = (−0.396, 0.308)]. These results were further supported by exploratory Bayesian statistics, which provided 2–6 times more evidence for *H_0_* over *H_1_* (small: *BF_10_^+^* = 0.60; large: *BF_10_^+^* = 0.16). For small search fields, the negative correlation between baseline performance and tDCS-related changes was once more reproduced, but again for both anodal and sham tDCS (anodal: ρ(29)=−0.67, *p_Bonf_* < 0.001; sham: ρ(29)=−0.54, *p_Bonf_* = 0.004), whereas for large search fields exploratory correlation analyses failed to reach significance in any condition (anodal: ρ(29)=−0.38, *p_Bonf_* = 0.069; sham: ρ(29)=−0.35, *p_Bonf_* = 0.107). These results were once more comparable when sensitivity *d’* or false alarm rates were used as dependent variables instead or when combining data across both experiments (for details see Supplementary material).

Our second experiment thus supports our previous null-effect despite left FEF stimulation and large search fields and renders alternative explanations for such null-effect (specifically right FEF stimulation and small search fields) rather unlikely.

### Blinding success

In both experiments, most participants correctly assigned stimulation types to test sessions (Experiment 1: 28/29 participants, 96.55%; Experiment 2: 26/31 participants, 83.87%). However, only 14/31 (45.16%; chance level at 50%) participants from Experiment 2 correctly identified the intended stimulation effect, suggesting that the above results should be largely unbiased by potential stimulation-related expectation effects on visual search performance.

## Discussion

Based on previous reports of tDCS-driven visual search improvements^8^ and related implications of tDCS as a potential therapeutic tool for the treatment of attention impairments,^6^ this study set out to test for a potential boost of such effect through right FEF stimulation and for a possible dependency on search field size. However, in contrast to previous literature^8^ and our hypotheses, none of our experiments provided any evidence for stimulation-specific visual search improvements through FEF tDCS. This null-effect was independent of stimulation site and search field size, rendering these factors as potential alternative explanations for the observed negative findings rather unlikely. It should be noted, though, that we cannot entirely exclude other small experimental differences (e.g. single-feature versus conjunction search with fewer versus more items) as potential explanations for diverging results since this study was no exact replication (even though we are not aware of a potential moderating function of any of said factors, which have further been balanced by shorter search times to equalize task difficulties, resulting in highly comparable performance scores and therefore similar chances for potential tDCS effects to arise). We can also not exclude potential effects of right FEF stimulation in combination with large search fields. However, if tDCS (whose application in the present experiments closely mirrored that of previous studies^8^) is truly supposed to be considered as general tool for cognitive enhancement and rehabilitation of visuo-spatial attention, such minor differences should constitute no obstacle for positive findings. Therefore, our results, while not entirely ruling out any tDCS-positive effects on attention or cognition more broadly, cast doubt on the broader generalizability of tDCS-related visual search improvements (at least for healthy young populations with well-tuned attention systems like ours).

This conclusion might not extend to elderly people or patients suffering from neglect or extinction, though, whose attention systems might be less-optimally tuned and who might thus possess greater potential for cognitive enhancement than our healthy young participants did. However, while we cannot exclude a potential stimulation-related attention improvement in such clinical and/or elderly populations, our results provide no support in favour of FEF tDCS as strong candidate for potential therapeutic tools for the treatment of attention disorders either. This is in line with previous inconsistencies in the tDCS literature,^20^ which have partly been attributed to differences in cognitive and/or physiological states at baseline,^13,14^ as well as to different inter-individual anatomies affecting electrical field spread.^21^ While such factors might have also contributed to the conflicting results observed in the present versus previous study,^8^ such inconsistencies highlight the susceptibility of tDCS effects, as well as the associated importance of proper baseline measurements (ideally not only behavioural but also physiological ones) and the potential of carefully (ideally individually) adjusted stimulation protocols (potentially through easily adoptable approximative measures like head circumference^22^). Another potential obstacle to consistent tDCS-related visual search improvements might lie in the method’s low spatial resolution: Single-cell FEF stimulation in monkeys has shown improved detection for stimuli within the cell’s receptive field but impairments for information outside of it,^23^ suggesting complex and potentially conflicting effects through widespread tDCS stimulation. Thus, whereas the present experiments do not support substantial visual search improvements through tDCS, FEF stimulation might prove fruitful for attentional improvements with spatially more fine-tuned methods. Therefore, additional research with alternative methods and clinical/elderly populations as well as systematic meta-analyses of such to help identify the mechanisms and conditions under which tDCS can or cannot support human cognition will be essential for the much aspired but currently limited treatment of attention impairments and clinical disorders more generally.

## Supplementary Material

fcaf480_Supplementary_Data

## Data Availability

The data and analyses that support the findings of this study are openly available on OSF at https://osf.io/f64h7.
