# Supplementary material for: Transcranial direct current stimulation over the frontal eye field has no effect on visual search performance
Source: Brain Commun. 2025 Dec 8;7(6):fcaf480. doi: 10.1093/braincomms/fcaf480 (PMC12709282; doi:10.1093/braincomms/fcaf480)
Supplement: fcaf480_Supplementary_Data [file fcaf480_supplementary_data.pdf]

Supplement

Control analyses

To exclude the possibility that the chosen dependent variable (i.e., accuracy) was not sensitive enough to detect potential tDCS-related visual search improvements, we repeated all original analyses from both experiments with the following two alternative dependent variables:

- Signal detection parameter sensitivity (*d'*) (i.e., the ability to distinguish target-present from target-absent trials computed as $z(H)$-$z(FA)$, where $z(H)$ and $z(FA)$ correspond to the z-transformed rate of correct target-present relative to all target-present trials and the z-transformed rate of incorrect target-absent relative to all target-absent trials, respectively)
- Signal detection parameter false alarm rates (i.e., the rate of incorrect target-absent relative to all target-absent trials)

All statistical tests were identical to the original analyses with the following two exceptions: 1) Violations of *t*-test assumptions (i.e., non-normality and/or outliers) were accounted for using additional Wilcoxon signed rank tests. 2) T-Test directionality was adjusted for the analysis of false alarm rates (i.e., expecting lower false alarm rates for anodal compared to sham tDCS).

To further leverage on the full data spectrum and to systematically compare stimulation sites (left vs. right FEF), in a third and last control analysis, we combined data from the common small-field condition of both experiments and analysed the baseline-corrected percentage of correct responses in a joint 2x2 mixed ANOVA with stimulation (anodal vs. sham) as within-subject factor and experiment/stimulation site (experiment one/right FEF vs. experiment two/left FEF) as between-subject factor. Bayesian test equivalents were used to quantify the evidence for the reported data under models including the above factors or their interactions compared to any other model without such (BF_{incl}). Given the presence of an outlier in one of the groups and variables of interest, both analyses (frequentist and Bayesian) were performed twice, once with and once without said outlier.

All control analyses were exploratory (i.e., not included in the preregistration) and were uncorrected for multiple testing (i.e., corrections for multiple, independent statistical tests were

only performed within individual control analyses similar to the original analyses, but not across control analyses).

Control results

In line with our initial conclusion of no tDCS-specific visual search improvements, none of the control analyses yielded a significant difference of baseline-corrected search performances between the two stimulation types (anodal vs. sham) independent of stimulation site (left vs. right FEF) and search field size (small vs. large fields). This was the case for the two control analyses with sensitivity *d'* and false alarm rates as alternative dependent variables (all $p \geq .229$; for details see Supplementary Table 1), as well as for the joint analysis of the two experiments with the percentage of correct responses as dependent variable (all $p \geq .145$; for details see Supplementary Table 2).

Bayesian statistics further supported these results by providing 3-7 times more evidence for the absence of a sensitivity or false alarm difference between anodal and sham tDCS ($0.14 \leq BF_{10} \leq 0.38$; for details see Supplementary Table 1). Bayesian results further suggested that the most likely model given the observed data was a null-/subject-only model, that this null-model was 3-5 times more likely than any other model ($3.11 \leq BF_{M\text{-}Null} \leq 4.54$) and that the probability of observing the presented data under any model that included the factors stimulation, experiment/stimulation site or their interaction was 2-17 times lower compared to any other model without the respective factors ($0.06 \leq BF_{incl} \leq 0.57$; for details see Supplementary Table 2).

Similarly, and in line with our conclusion of no tDCS-specific baseline dependencies, significant negative correlations between baseline performance (sensitivity/false alarm rates) and tDCS-related visual search improvements were either equally present in both stimulation conditions (anodal and sham) or not at all (for details see Supplementary Table 1). Together, these control analyses suggest no systematic influence of stimulation type (anodal vs. sham tDCS) or stimulation site (left vs. right FEF) on visual search performance and provide further support for our original null-findings

Supplementary Table 1

Summary of results for the first two control analyses (sensitivity/false alarm rates; rows) and experiments/conditions (columns)

Analysis	Measure	Exp 1: Right FEF, small fields	Exp 2: Left FEF, small fields	Exp 2: Left FEF, large fields
**Sensitivity d-prime**	**Mean ± SD**	A_{base}: 1.96±0.49, A_{peri}: 2.09±0.62<br>S_{base}: 2.00±0.63, S_{peri}: 2.06±0.68	A_{base}: 2.19±0.73, A_{peri}: 2.37±0.61<br>S_{base}: 2.19±0.73, S_{peri}: 2.24±0.58	A_{base}: 1.88±0.71, A_{peri}: 2.01±0.67<br>S_{base}: 1.61±0.61, S_{peri}: 1.82±0.68
	**Student *t* (Wilcoxon)**	$t(28)=0.31$, $p=.378$, $d=0.058$, $CI_{95}=[-0.307, 0.422]$, $BF_{10}^{+}=0.26$<br>$W=195.00$, $p=.688$, $r_{rb}=-0.10$	$t(30)=0.75$, $p=.229$, $d=0.135$, $CI_{95}=[-0.220, 0.488]$, $BF_{10}^{+}=0.38$<br>/	$t(30)=-0.50$, $p=.691$, $d=-0.091$, $CI_{95}=[-0.443, 0.263]$, $BF_{10}^{+}=0.14$<br>/
	**Spearman Correlation**	A: $\rho(27)=-0.41$, $p_{Bonf}=.028$<br>S: $\rho(27)=-0.41$, $p_{Bonf}=.026$	A: $\rho(29)=-0.66$, $p_{Bonf}<.001$<br>S: $\rho(29)=-0.64$, $p_{Bonf}<.001$	A: $\rho(29)=-0.37$, $p_{Bonf}=.083$<br>S: $\rho(29)=-0.30$, $p_{Bonf}=.197$
**False alarm rates**	**Mean ± SD**	A_{base}: 0.09±0.06, A_{peri}: 0.09±0.08<br>S_{base}: 0.09±0.08, S_{peri}: 0.10±0.11	A_{base}: 0.08±0.11, A_{peri}: 0.07±0.10<br>S_{base}: 0.07±0.08, S_{peri}: 0.06±0.07	A_{base}: 0.09±0.09, A_{peri}: 0.09±0.10<br>S_{base}: 0.12±0.10, S_{peri}: 0.11±0.10
	**Student *t* (Wilcoxon)**	$t(28)=-0.48$, $p=.316$, $d=-0.090$, $CI_{95}=[-0.454, 0.276]$, $BF_{10}^{-}=0.30$<br>$W=167.50$, $p=.559$, $r_{rb}=0.03$	$t(30)=0.19$, $p=.574$, $d=0.034$, $CI_{95}=[-0.318, 0.386]$, $BF_{10}^{-}=0.17$<br>$W=131.50$, $p=.571$, $r_{rb}=0.04$	$t(30)=0.36$, $p=.641$, $d=0.065$, $CI_{95}=[-0.288, 0.417]$, $BF_{10}^{-}=0.15$<br>$W=214.00$, $p=.730$, $r_{rb}=0.13$
	**Spearman Correlation**	A: $\rho(27)=-0.26$, $p_{Bonf}=.167$<br>S: $\rho(27)=-0.13$, $p_{Bonf}=.510$	A: $\rho(29)=-0.57$, $p_{Bonf}=.002$<br>S: $\rho(29)=-0.58$, $p_{Bonf}=.001$	A: $\rho(29)=-0.18$, $p_{Bonf}=.662$<br>S: $\rho(29)=-0.43$, $p_{Bonf}=.034$

Note: Exp 1 = experiment one; Exp 2 = experiment two; FEF = frontal eye field; A_{base} = anodal stimulation, baseline assessment; A_{peri} = anodal stimulation, peri assessment; S_{base} = sham stimulation, baseline assessment; S_{peri} = sham stimulation, peri assessment; A = anodal condition; S = sham condition.

Supplementary Table 2

Summary of results for the third control analysis (joint percentage correct)

Frequentist effects	Mean ± SE & Test statistics
**Stimulation**	A: +1.48±0.78 (+1.46±0.79)<br>S: -0.23±0.94 (-0.57±0.89)<br>F(1, 58 (57))=1.52 (2.19), p=.223 (.145), η^2_p=.026 (.037)
**Exp/FEF**	Exp1/rFEF: +0.59±0.74 (+0.59±0.70)<br>Exp2/lFEF: +0.66±0.72 (+0.30±0.69)<br>F(1, 58 (57))=0.01 (0.09), p=.942 (.771), η^2_p=.000 (.002)
**Stimulation × Exp/FEF**	A × Exp1/rFEF: +1.31±1.11 (+1.31±1.12)<br>S × Exp1/rFEF: -0.14+1.36 (-0.14±1.27)<br>A × Exp2/lFEF: +1.65±1.08 (+1.60±1.10)<br>S × Exp2/lFEF: -0.32±1.31 (-1.00±1.25)<br>F(1, 58 (57))=0.04 (0.18), p=.852 (.676), η^2_p=.001 (.003)

Bayesian models	P(M)	P(M│data)	BF_M	BF_{10}
**Null model (incl. subject)**	0.20 (0.20)	0.53 (0.44)	4.54 (3.11)	1.00 (1.00)
**Stimulation**	0.20 (0.20)	0.28 (0.36)	1.53 (2.21)	0.52 (0.81)
**Exp/FEF**	0.20 (0.20)	0.12 (0.10)	0.53 (0.44)	0.22 (0.23)
**Stimulation + Exp/FEF**	0.20 (0.20)	0.06 (0.08)	0.25 (0.36)	0.11 (0.19)
**Stimulation + Exp/FEF + Stimulation × Exp/FEF**	0.20 (0.20)	0.02 (0.02)	0.06 (0.10)	0.03 (0.05)

Bayesian effects	P(incl)	P(incl│data)	$BF_{Inclusion}$
**Stimulation**	0.60 (0.60)	0.35 (0.46)	0.36 (0.57)
**Exp/FEF**	0.60 (0.60)	0.19 (0.21)	0.16 (0.17)
**Stimulation × Exp/FEF**	0.20 (0.20)	0.02 (0.02)	0.06 (0.10)

Note: Values outside vs. inside of brackets correspond to analyses with vs. without outlier, respectively. Exp/FEF = experiment/frontal eye field; A = anodal stimulation; S = sham stimulation; Exp1/rFEF = experiment one, right frontal eye field stimulation; Exp2/lFEF = experiment two, left frontal eye field stimulation; A × Exp1/rFEF = anodal stimulation over the right frontal eye field; *S × Exp1/rFEF* = sham stimulation over the right frontal eye field; *A × Exp2/lFEF* = anodal stimulation over the left frontal eye field; *S × Exp2/lFEF* = sham stimulation over the left frontal eye field.
